# Can money buy health? Using a natural experiment to guide interventions to address the socioeconomic inequalities in health

**DOI:** 10.3389/fpubh.2024.1397127

**Published:** 2024-05-28

**Authors:** David Füller

**Affiliations:** ^1^Brandenburg Medical School, Brandenburg, Germany; ^2^Division of Cardiology, Nephrology, and Pulmonology, Department of Internal Medicine, University Hospital Brandenburg an der Havel, Brandenburg Medical School, Brandenburg, Germany

**Keywords:** socioeconomic factors, natural experiment, social determinants of health, citizen income, health outcomes

## Background

In January 2024, the Citizen's Income, a secured payment for people without employment in Germany, has increased by about 12%. This has caused a recent political and social debate because the raise will be significantly higher than the increase in wages and inflation, which as of February 2024 is 2.5% in Germany, the lowest level since June 2021.

## An epidemiological and social science perspective

Over the last decades, inequalities in health related to socioeconomic factors including the level of education and income have been highly persistent in Germany, Europe, and in the U.S. (1–3). In particular, a trend of widening disparities in life expectancy by income, associated with an increasingly strong association between low income and poor health, has become evident (2, 3). A disproportional increase of financial resources, e.g., for those receiving the Citizen's Income, can be used as a natural experiment for research purposes which provides the opportunity to examine the health-relevant effects of a significant increase in individual purchasing power. Whether attempting to close the widening gap in financial income in a natural experiment can alleviate disparities in health constitutes a major interest of global public health. In Germany, such natural experiments could be examined using health monitoring data.

Using natural experiments to investigate the significance of the social determinants of health for health-related outcomes is interesting because they can establish causal relationships. In previous work, the causal effects of education on health outcomes in the UK have been assessed using data of the UK Biobank (4). The authors found consistent evidence that raising the minimum school leaving age in the UK in 1972 had beneficial effects on diabetes incidence and mortality risk.

Providing insights from longitudinal studies and natural experiments which focus on the association of socioeconomic determinants and health outcome measures can guide health policy and public health interventions because they can evaluate policy changes and the success or failure of previous interventions. Therefore, they should include participant-level information on geographical segregation, adverse neighborhood environments, missing opportunities to eat healthy and exercise regularly, duration of unemployment, adverse occupational conditions, and facing discrimination based on sex, age, ethnicity, or poverty, among others, which are associated with an increased risk for ill health.

## A clinical perspective

To address socioeconomic inequalities in medicine, along with epidemiological research and public health initiatives, clinical interventions and research evaluating their effects are overdue. Low socioeconomic status is recognized to be a significant risk factor for ill health for many years, however, interventions to mitigate its associated increased risk in adverse health outcomes are rare (5). Previous studies have indicated that the higher all-cause mortality risk associated with lower socioeconomic status is comparable to that of physical inactivity, high alcohol intake, obesity, depression, and high cholesterol levels (6, 7). In a subgroup analysis of a randomized clinical trial, it has been shown that health interventions might be particularly effective in patients with lower socioeconomic status (8). For those involved in clinical trials, it is time to plan interventions to avert adverse health outcomes especially in those known to be at increased risk through focus on access to care appropriate to the needs of special populations, reducing barriers in healthcare, improved patient navigation and support, and engagement to collaborate with non-healthcare institutions for the benefit of patients and communities.

## Author contributions

DF: Conceptualization, Funding acquisition, Investigation, Validation, Writing – original draft.
